# Estimation of air pollutants emission (PM_10_, CO, SO_2_ and NO_x_) during development of the industry using AUSTAL 2000 model: A new method for sustainable development

**DOI:** 10.1016/j.mex.2019.06.010

**Published:** 2019-06-14

**Authors:** Ali Atamaleki, Saeed Motesaddi Zarandi, Yadolah Fakhri, Ehsan Abouee Mehrizi, Ghasem Hesam, Meysam Faramarzi, Mahdiyeh Darbandi

**Affiliations:** a Student Research Committee, School of Public Health and Safety, Shahid Beheshti University of Medical Sciences, Tehran, Iran; b Department of Environmental Health Engineering, School of Public Health and Safety, Shahid Beheshti University of Medical Sciences, Tehran, Iran; c Department of Environmental Health Engineering, Faculty of Public Health, North Khorasan University of Medical Sciences, Bojnurd, Iran; d North Khorasan University of Medical Sciences, Bojnurd, Iran; e Azad University of Tonekabon, Tonekabon, Iran

**Keywords:** A new method for estimation of industries development on air pollutant emission by modeling, Air pollution, Modeling, AUSTAL 2000, Cement factory, Industry development

## Abstract

There is well-documented relationship between industrial development and environmental pollution, but there are no enough studies that have predicted development impacts on pollutants emission. In the current study, impacts of three development periods of Bojnourd cement factory on pollutants emission (CO, SO_2_, NO_x_, and PM_10_) were investigated using the AUSTAL 2000 model. The collected emission data during 19 years were classified for each period and analyzed via the model, separately. Two sets of monitoring point (each contains 5 points) determined at the model; first set for estimation of pollutants concentration in residential areas (three villages, one suburban, and one city), and the second set for model validity assessment which located near the factory.

• According to model results, the second development period had the highest emission load per unit area for PM_10_ and SO_2_ by 164% and 262%, respectively. However, by applying the bag filter at the beginning of the third period, SO_2_ and PM_10_ concentrations were reduced significantly to the same as the first period.
• Unlike the two previous pollutants, emissions load of NO_x_ and CO per unit area were increased in both the second period (167% and 154%, respectively) and third period (182% and 337%, respectively). Moreover, the model showed a good agreement compared with the field measured data that it could be usable to predict pollutants emission.
• The findings of this paper prove the predicting importance of the emissions prior to construction or any stages of industries upgrading and development. In other words, it emphasizes environmental protection during economic boost to maintain harmony between nature and sustainable development. Also, the model showed how the use of pollution control equipment (bag filter) during development can be effective to reduce the pollutants emission.

## Graphical abstract

**Figure:**
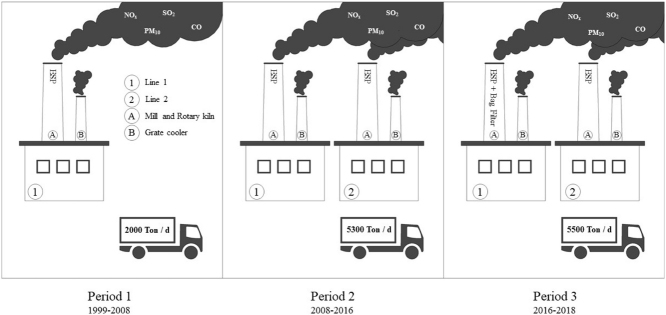

**Specifications Table**

**Table:** 

Subject area:	Environmental Science
More specific subject area:	Air pollution monitoring
Method name:	A new method for estimation of industries development on air pollutant emission by modeling.
Name and reference of original method:	Air pollution modeling
Resource availability:	NA

## Method details

There is a well-established correlation between economic progress and environmental contamination [1,2]. A survey done by Al-Mulali et al. demonstrated that CO_2_ production and GDP growth are two cointegrated factors [3]. The world’s economic growth and the development of industries by increasing the products and consumption rate have led to an increase in industrial emissions to the atmosphere [[4], [5], [6], [7]]. Cole et al. (2011) surveyed 112 major cities in China and reported that most air and water contaminants had a direct relationship with economic growth [8].

Over the past 50 years, the effects of air pollution on public health has been a major concern [9]. Several studies have confirmed the relationship between air pollutants (PM_s_, CO, SO_2_, and NO_x_) and health-related problems including respiratory and cardiovascular disorders, blood pressure, and lung cancer, which lead to the people deaths [10]. According to the World Health Organization (WHO) report, 2.4 million people around the world die per year due to air pollution [11]. In this regard, a study on air pollution in Mashhad metropolis, Iran, showed about 1800 death [12]. Also, the negative impacts of air pollution on animals, plants, water ecosystem, and other objects have been proven [13].

Nowadays, cement production has an intense progress. This industry as one of the most energy-consuming sectors emits high levels of pollutants into the air [14]. Iran is one of the top 15 cement producers of the world and production developing has been the priority of the cement industry in the last decade. Following this development, the contamination level also has increased [15]. Pollutants such as NO_x_, SO_2_, PM_s_, CO, and CO_2_ are the most commonly released substances from these industries [16,17]. During cement production, PM_s_ are typically generated from mechanical processes. Other gases are often produced by fuel combustion [18]. Dispersion of such contaminants from high level stacks has a significant impact on their emissions at the ground surface around the factory [19,20].

Modeling is an efficient and reliable method for forecasting and simulating the pollutants dispersion [[21], [22], [23]]. Dispersion models have advantages such as long and short-term estimates of contaminants at specified monitoring points and calculation of dispersion distance [24]. AUSTAL View is a steady-state Lagrangian dispersion model that has a graphical and ergonomic user interface for AUSTAL 2000 [25,26]. In the investigation done by Lutman et al., Lagrangian model for long-range prediction of radionuclides provided more reliable results than the Gaussian model [27]. This simulator can predict the distribution of pollutants and odor from the point, line, and area sources in flat or elevated terrain option. Moreover, AUSTAL View is capable to create a Wind-rose and analysis of meteorological parameters [28,29]. According to a study conducted by Paas and Schneider, AUSTAL 2000 showed more valid results than ENVI-met [28]. Moreover, another literature proved the higher validity of AUSTAL 2000 than CFD (ANSYS CFX v14) and VDI guideline 3783 [30].

The changes of source properties, weather conditions, and terrains can affect the pollutants dispersion. So, development and changes of the process can be effective on the simulation outcomes [14,19,31]. The impacts of the construction and development of industry on air pollutants emission should be investigated prior to starting up. Air pollution modeling provides predicting the impact of these changes on the distribution of pollutants which leads to form a stable relationship between human activities and natural worlds. Accordingly, this study follows the three objectives by the model: (1) how the dispersion of air pollutants by cement factory, (2) changes of pollutant emission during the factory development periods, and (3) effects of pollution control equipment on pollutants emission.

## Study area

**Fig. 1:**
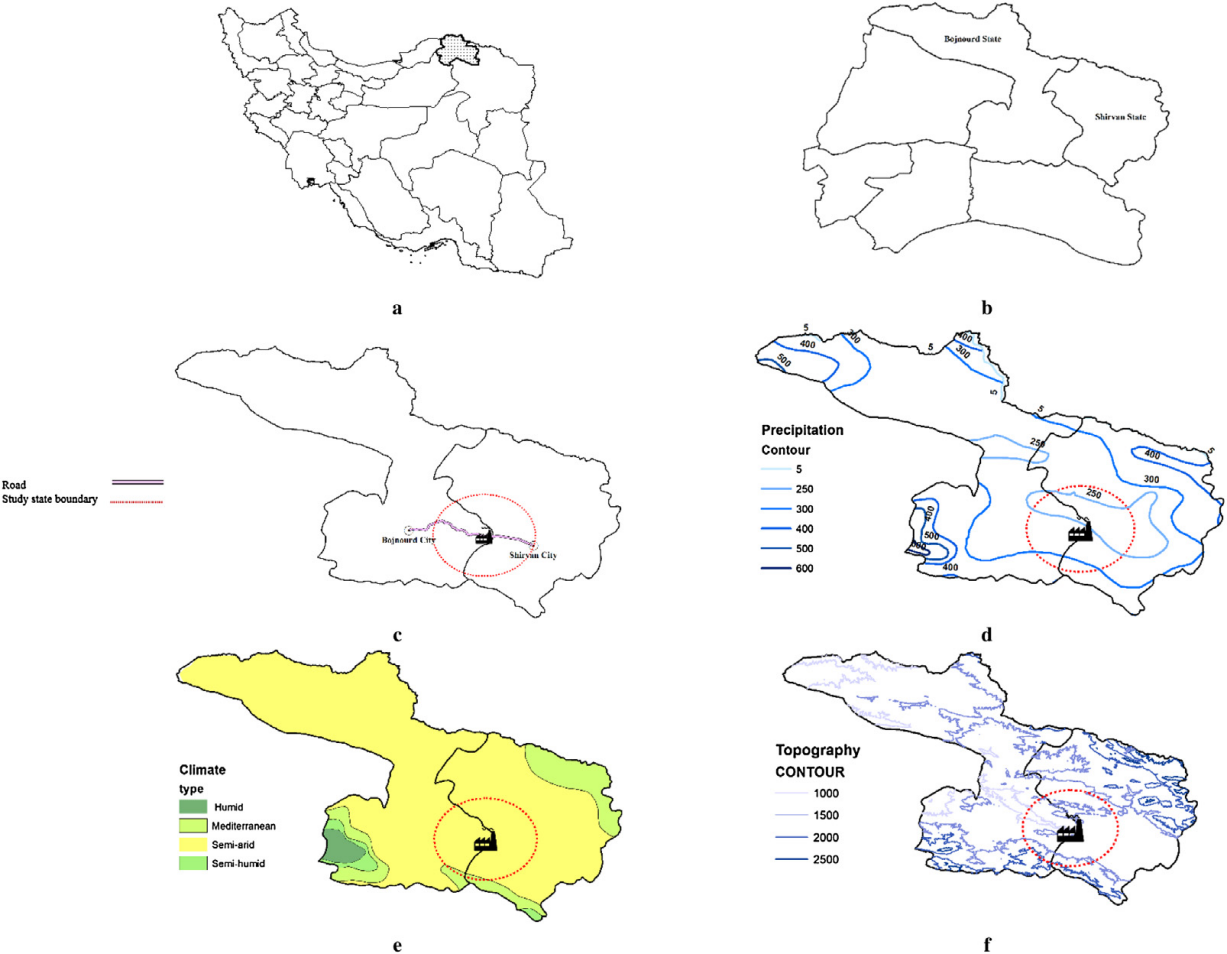
a) North-Khorasan province location, b) Bojnourd and Shirvan States, c) Cement factory location and study area, d) Precipitation map, e) Climate map, and f) Topography map.

Bojnourd is the capital city of North-Khorasan Province in the northeast of Iran. Cement factory is located in 37 km southeast of this city (37°27′N and 57°41′E), near Shirvan city (Fig. 1). The factory is surrounded by two mountain ranges and has 60 villages in a 20 km radius round it. According to Fig. 1, the climate condition of the study area is semi-arid and annual precipitation range is 250–300 mm. The elevation of this area is about 1000–2000 m above sea level.

## Materials and methods

### Source characteristics and development periods

**Fig. 2:**
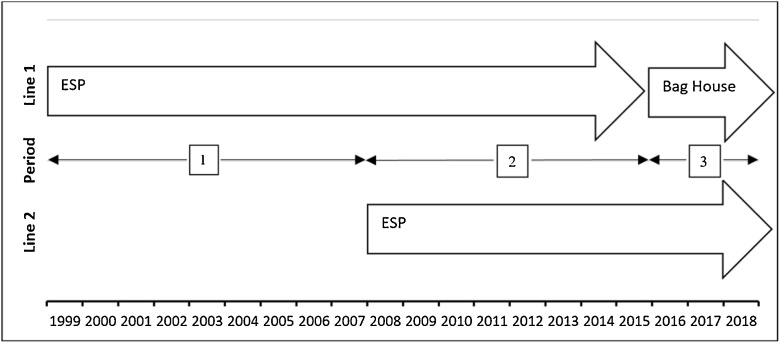
Schematic diagram of Bojnourd cement factory development.

In this study, four main point sources with steady-state emission rate within two production line were considered. The first line of the Bojnourd cement factory with the nominal capacity of 2000 tons per day was launched in 1999. An ESP for control of generated particles was installed in this line (source 1). To increase production, the second line (nominal capacity of 3300 tons per day) with another ESP was launched (source 3) in 2008. In 2016, in addition to increasing the capacity of 200 tons per day at line 1, for pollution control improvement, one bag filter was added to former ESP. Totally, this factory produces 5500 tons per day, which is in accordance with the recommended values (4000–7000 tons per day) [32]. It is noteworthy that the consumed fuel of factory in all periods and units has been natural gas. Physical information of stacks and a schematic diagram of the factory’s development are presented in Table 1 and Fig. 2, respectively.

**Table 1:** Physical characteristics of sources.

	Source	Unit Processing	Location (m)		Diameter (m)	Height (m)	Temperature (C)	Flow Velocity (m/s)
			Longitude (E)	Latitude (N)				
Line 1	1	Mill and Rotary kiln	40295804.02	4147820.65	3.6	115	108	26.1
	2	Grate cooler	40295804.02	4147820.65	3	30	200	19.7
Line 2	3	Mill and Rotary kiln	40295853.17	4147819.45	3.25	96	92	17.2
	4	Grate cooler	40295853.17	4147819.45	3	30	215	7.1

### Data collection

In our research, four main pollutants of the cement industry (CO, NO_x_, SO_2_, and PM_s_) [6] were investigated. According to Zhang et al., PM_10_ accounts for more than 50% of the total particles in the cement industry exhaustion [33]. Therefore, PM_10_ was considered as the representative of PM_s_. Calibrated TESTO 350 device was applied to determine the concentration of gases such as CO, SO_2_, and NO_x_; and, calibrated TES 5200 device was applied for dust detection. For ambient air monitoring, two devices were used: TSI 8520 for particles (PM_10_) and AeroQual series 200 for gases (CO, SO_2_, and NO_x_). To measure physical characteristics such as velocity, TESTO 512 device was used.

Hourly meteorological data including wind speed, wind direction (36 categories), temperature, and precipitation over the 5 past years (2012–2017) were collected from Shirvan meteorological station, which is located up to 15 km from the factory.

### Model setup

Input data such as encoded meteorological parameters, terrains as SRTM3 format (Global ˜ 90 m) and source characteristics (according to Table 1) were added to the model. The modeling grid was placed at sources downwind as single squares (22.6 km × 22.6 km) with the resolution of 200 m. To monitor the pollutants concentration (by hourly and daily means assessment), a set of points in residential areas (three villages, one suburban, and Shirvan city) were determined in study area. Also, to assess the model’s validity, the collected ambient air monitoring data recorded for 8 months at 5 points (second set) near the factory were compared with model results at the same locations. For this purpose, after providing the normal distribution of data with the Kolmogorov-Smirnov test, the distribution of data did not show a significant difference with a normal distribution (P value > 0.05). Therefore, the Pearson correlation coefficient was used. Characteristics of the monitoring points are shown in Table 2. The residential structures were not included in the model. In the direction of the prevailing wind, the monitoring points were placed on the interior of each residential area.

**Table 2:** Characteristics of monitoring points.

Monitor point		Location (m)		Height (m)	Distance of source (m)	Description
		Latitude	Longitude			
Model monitoring	1	565371.4	4144931	1.5	4687	Reza Abad village
	2	566618.02	4145901.8	1.5	5881	Bigan village
	3	568978.88	4144180.8	1.5	8304	Tudeh village
	4	577727.29	4144897.9	1.5	16987	Ziarat suburban
	5	580442.65	4141685.9	1.5	20048	Shirvan city
Ambient air monitoring	6	560586.81	4145168.6	1.5	224	South of sources
	7	559945.13	4145564.6	1.5	842	West of sources
	8	560633.03	4145569.6	1.5	274	North of sources
	9	561050.91	4145541.8	1.5	375	North- east of sources
	10	560316.33	4145197.4	1.5	453	South- west of sources

After running the model, dispersion patterns of pollutants as contour lines (height of 0–3 m) were obtained for three periods, separately. The affected areas of them for each pollutant was calculated by Arc GIS 10.2 software and compared together using the weighted average surface of pollutants load [34]. The weighted average surface was assumed as the contamination load per area for pollution comparison between periods.

## Results and discussion

### Meteorology

**Fig. 3:**
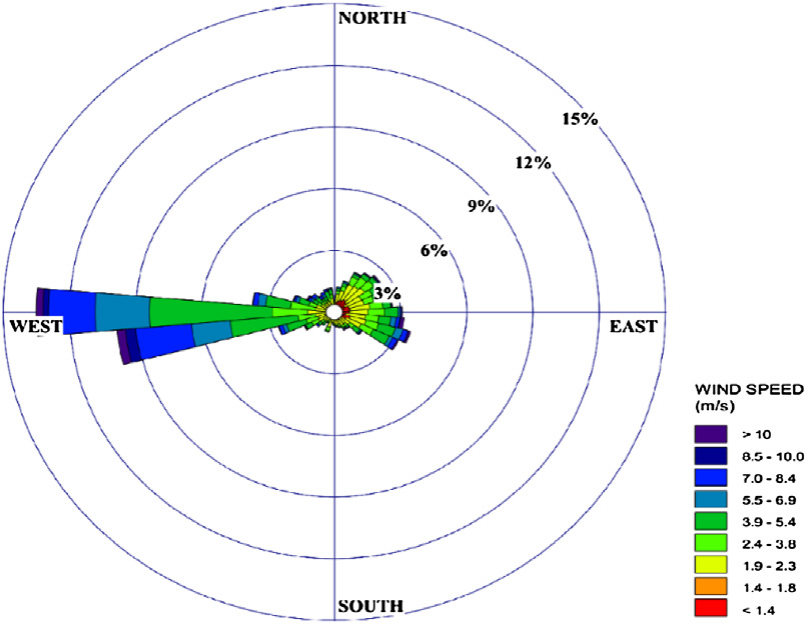
Windrose plot.

Drawn wind-rose shows the frequency of wind direction and speed in the study area (Fig. 3). According to the wind-rose, the local wind speed is 2.92 ± 2.41 m/s; the most frequent wind direction is 13.47%, which blows from 264–275°; and the most frequent dispersion class is an unstable condition (IV), 39.2%. The seasonal rank order of average wind speed was summer (3.56 m/s) > spring (3.50 m/s) > autumn (2.64 m/s) > winter (1.92 m/s).

### Development effects on source emission

**Fig. 4:**
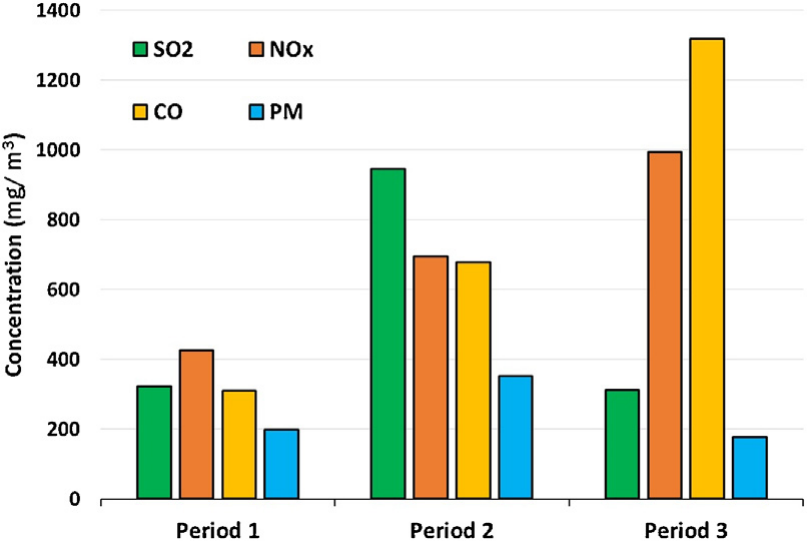
Variation of source output along three development period.

Fig. 4 presents the mean concentration of pollutants at three periods of factory development. The maximum output concentration of PM_10_ and SO_2_ were 944.8 mg/m^3^ and 352.6 mg/m^3^, in the second period, respectively. Also, in the third period, the maximum output concentration of NO_x_ and CO was obtained 994 mg/m^3^ and 1318.1 mg/m^3^, respectively. As noted earlier, primary mechanical units including mills and blenders, which use electricity as power supply, are responsible for the release of particulates. Processor units (calciner and kiln) that use fossil fuels contribute to the production of a portion of the output gases [35]. Usually, CO is released due to incomplete fuel combustion. SO_2_ generation can be attributed to both fuel and raw material properties. In addition to fuel property, NO_x_ can also be of a thermal type [25,36,37]. After the establishment of the second line and the increased production capacity, the generation of all pollutants increased significantly. Hence, the SO_2_ and CO concentrations release more than twice. However, in the third period, when a bag filter in line 1 was installed to control the PM_10_, a significant reduction occurred in both SO_2_ and PM_10_; but, CO and NO_x_ increased again. CaO is one of the main components of cement that formed by the heat from the limestone [38,39]. According to following reactions (1 and 2), limestone can adsorb the SO_2_ and create CaSO_4_ [37,40]. Hence, the portion of Sulfur particulates (CaSO_4_) was filtered together with the PM_10_ in the bag filter during the third period. Although a study showed that NO_x_ at high concentrations of SO_2_ can be further removed in the bag filters [41], Nielsen et al. reported that an increase in temperature and redox alternation can increase the emission of SO_2_ [37]. Hence, it can be concluded that the decadence of combustion conditions during development periods can be a major factor in increasing CO, NO_x_, and SO_2_.

(1) CaCO_3_ (s) ↔ CaO (s) + CO_2_ (g)

(2) CaO (s) + SO_2_ (g) + 1/2 O_2_ (g) ↔ CaSO_4_ (s)

The rank order of air contaminants based on their concentration in period 1 was NO_x_ > SO_2_ > CO > PM_10_; period 2, SO_2_ > NO_x_ > CO > PM_10_; and period 3, CO > NO_x_ > SO_2_ > PM_10_. The rank order of periods based on concentration of SO_2_ was period 2 > period 1 > period 3; NO_x_, period 3 > period 2 > period 1; CO, period 3 > period 2 > period 1; and PM_10_, period 2> period 1 > period 3 (Fig. 4). The origin of SO_2_ emissions in cement stacks output is mostly via the oxidation of existing pyrite and Fe_2_S in the cement raw materials [37,42,43]. So, the more increase in SO_2_ concentration than NO_x_ in the second period can be attributed to the production capacity extension than fuel combustion during production. CO increasing over these three periods can be due to the incomplete fuel combustion and inefficient processes of factory.

Based on the results (Fig. 1 Supplementary), NO_x_ (same as CO) had the highest emission load per unit area during the third period. The rank order of periods based on the weighted mean concentration of NO_x_ and CO in the predicted covering area was Period 3 > period 2 > Period 1, which can be attributed to the increase in factory production during three periods. It can also be probable that the development policy of the factory did not lead to an energy-saving and pollutants reduction in the release of these two gases [44]. Increased carbon emissions in some of China's cities along with industrial development was observed which reduced by modification of energy intensity and structure [45]. Nevertheless, many provinces of China have significant differences in terms of development and environment protection [46]. The highest concentration of PM_10_ and SO_2_ were obtained in the second period and reduced after utilization of the bag filter at line 1 (Source 3). The rank order of periods based on the concentration of PM_10_ was period 2 > period 1 ˜ period 3; and for SO_2_, it was period 2 > period 3 > period 1.

The maximum coverage area of NO_x_ was observed in the range of 0.2–0.5 μg/m^3^ at period 1; 0.2–0.5 μg/m^3^ at period 2; and 0.5–1 μg/m^3^ at period 3. For PM_10_, the predicted maximum coverage area during period 1 was at the range of 0.01–0.05 μg/m^3^; period 2, 0.05–0.1 μg/m^3^; and period 3, 0.01–0.05 μg/m^3^. The maximum predicted coverage of SO_2_ during period 1, period 2, and period 3 was 0.2–0.5 μg/m^3^. Also, for CO, this coverage was 0.2–0.5 μg/m^3^ during period 1 and period 2; and 2–5 μg/m^3^ during period 3.

### Monitoring points

As mentioned above, 5 monitoring points at residential areas were determined for assessment of predicted concentrations by model. Since seasonal variations of sources output were not considered in the model input, the changes in pollutant concentration in monitoring points for each period can be attributed to environmental conditions such as meteorological and geographical properties. Generally, in atmospheric modeling, with an increase in the distance from sources, the pollutants concentrations due to dilution effect decreased [47,48]. Based on Fig. 2 Supplementary, in all developing periods, by increasing the distance from the sources (MNT-1 to MNT-5), hourly mean concentration of all pollutants were reduced significantly (P < 0.001). Based on the predicted results at 5 residential monitoring points, all hourly calculated concentrations were lower than primary and secondary National Ambient Air Quality Standards (NAAQS) [49].

### Model validity

According to the results, most of the observed concentrations of pollutants are higher than the predicted ones. This discrepancy can be attributed to background concentration, which was ignored in our research. Hybrid simulation – composed of Lagrangian (AUSTAL 2000), meteorological, and a chemical-transport Eulerian model, showed that the observed annual and hourly mean concentrations were higher than a predicted concentration, probably due to urban morphology characteristics [50]. As mentioned earlier, the Pearson correlation coefficient was used for the model validity assessment. Based on the quantile-quantile plot (Fig. 3 Supplementary), there are not any significant differences between predicted and observed concentrations at MNT-6 for PM_10_, SO_2_; MNT-7 for SO_2_, CO, and NO_x_; MNT-8 for PM_10_, SO_2_, and NO_x_; MNT-9 for PM_10_, SO_2_, CO, and NO_x_; MNT-10 for PM_10_, SO_2_, and NO_x_. Moreover, the rank order of pollutants validity was SO_2_ (5 point) > PM_10_ (4 point) = NO_x_ (4 point) > CO (2 point). AUSTAL 2000 model had a good agreement with the prediction of pollutants concentration at determined monitoring points. Nevertheless, Oettl et al. validated Gaussian and Lagrangian models for NOx dispersion on a road and concluded that Lagrangian model had a better agreement than Gaussian (with the index validity of 0.81–1) [51].

## Conclusion

Economic growth and development have always been accompanied by an increase in energy consumption and environmental pollution. This issue highlight the aim of sustainable development which is to balance our economic, environmental and social needs. In this paper, the effects of three developing periods of Bojnourd cement factory on pollutants dispersion during 19 years were investigated. Subsequently, the concentration of dispersed pollutants around the factory, especially at five residential points were predicted and compared for each period using AUSTAL 2000 model. Also, the validity of the model by comparison of field measurement and model results by the second set of monitoring points near the factory was investigated.

Based on the results of this study, the second development period plays an important role in the production and dispersion of the pollutants to the atmosphere, when production capacity was upgraded to 260% higher than prior. As a result, corresponding rises of 219% in CO, 163% in NO_x_, 292% in SO_2_, and 178% for PM_10_ occurred. With an increase in production capacity to 103% at the beginning of third-period and applying bag filter at line 1, SO_2_ and PM_10_ were reduced the same level as in period 1 (33% and 50%). However, no policy has been considered to control the gases in each of the three periods. The monitoring data showed that the mean concentration of all pollutants was lower than the threshold concentration. Moreover, model output generally had a good agreement for all pollutants. SO_2_ had the highest validity compared to other pollutants at ambient air monitoring sites.

As noted earlier, application of the bag filter had a significant role in reduction of the particulate pollutants load per unit area. Considering that Iran is one of the top cement producer in the world, it is recommended that central government allocate subsidies to such industries for applying and upgrading their pollution control equipment, leading to balanced industry development. Finally, it can be concluded that industries’ growth and development have an important role in environmental pollution. Due to the direct impact of the source pollutants emission on around receptors, the prediction of the industrial development effects on environment and health is emphasized. In this regards, the use of modeling was proposed to predict the pollution level during development. Also, the use of upgraded and appropriate processing equipment along with appropriate management is recommended for improving the combustion conditions, reducing emissions and preventing environmental side effects.

## Declaration of Competing Interest

There is no conflict of interest.
