# Supplementary material for: Estimation of air pollutants emission (PM10, CO, SO2 and NOx) during development of the industry using AUSTAL 2000 model: A new method for sustainable development
Source: MethodsX. 2019 Jun 14;6:1581–90. doi: 10.1016/j.mex.2019.06.010 (PMC6612795; doi:10.1016/j.mex.2019.06.010)
Supplement: Supplementary file 1 [file mmc1.docx]

|  | **Period 1** | | | **Period 2** | | | **Period 3** | | |
| --- | --- | --- | --- | --- | --- | --- | --- | --- | --- |
| **NOx** (µg/m^3^) | 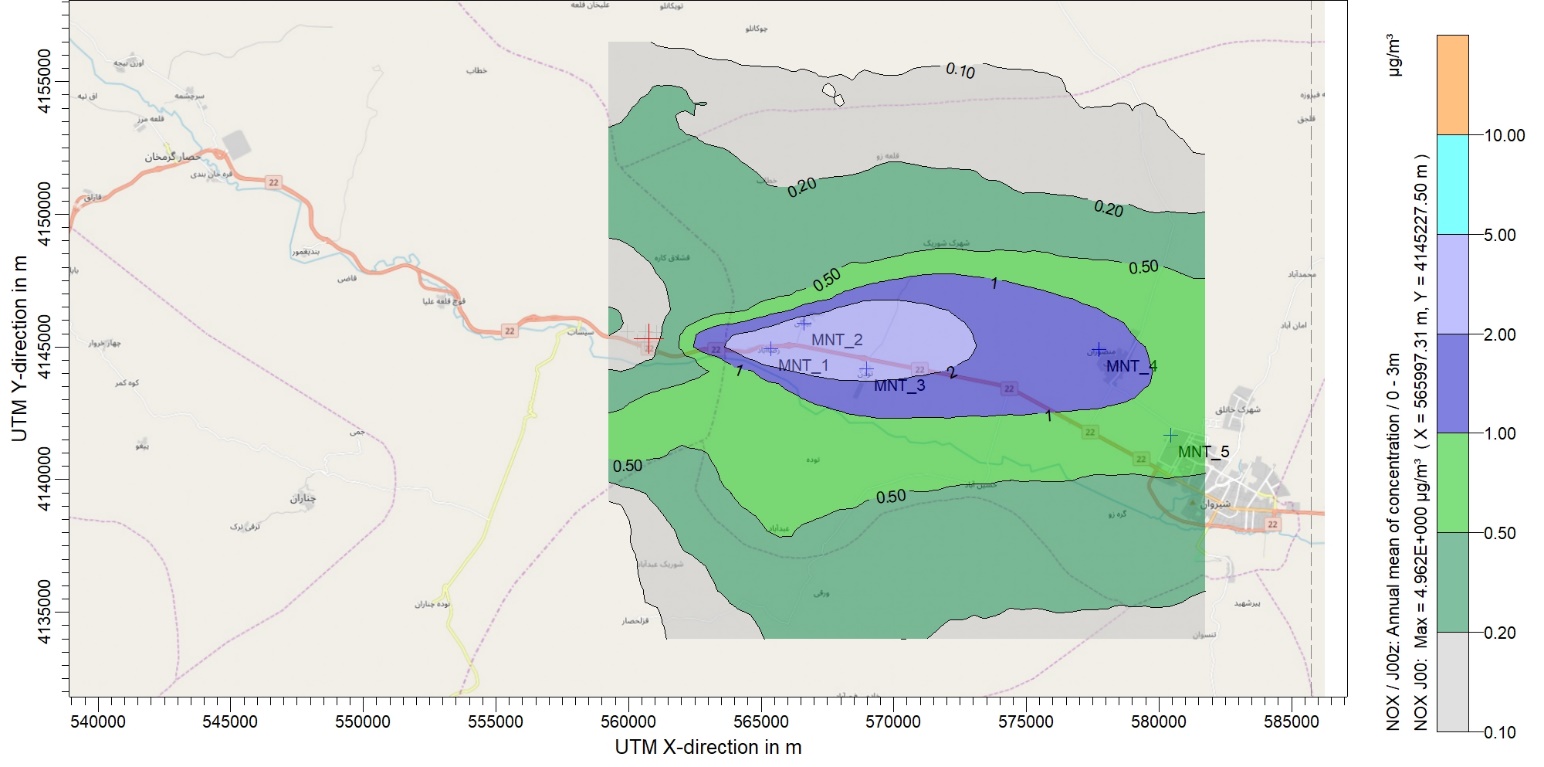 | | | 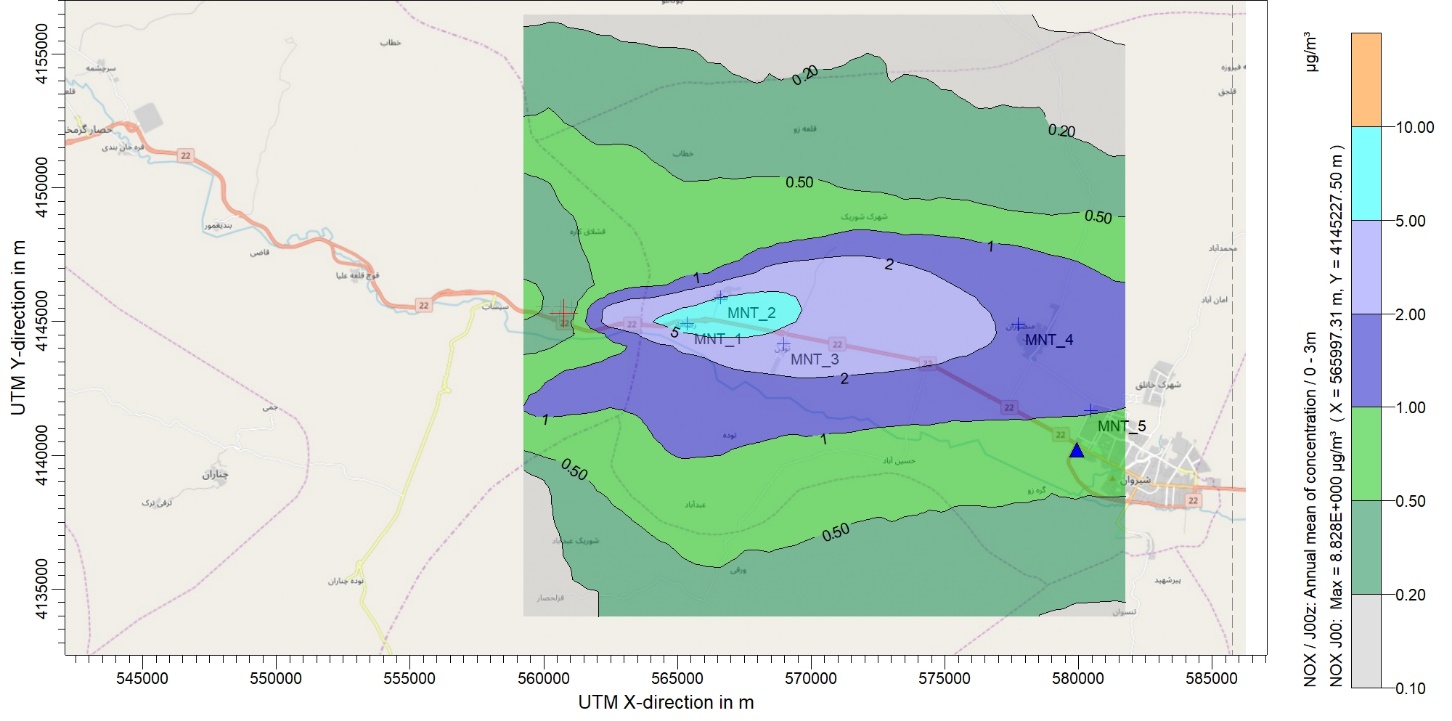 | | | 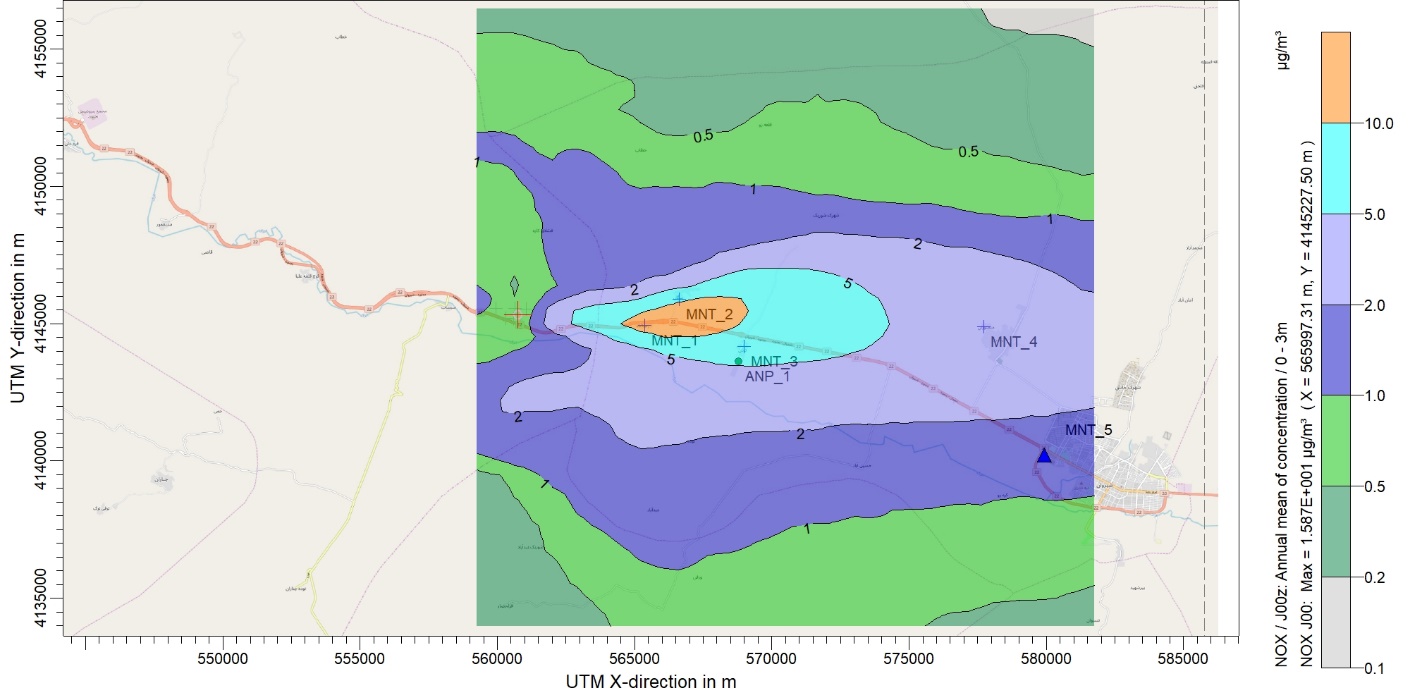 | | |
|  | Min= 0.05, Max= 4.96 | | | Min= 0.08, Max= 8.8 | | | Min= 0.15, Max= 15.8 | | |
|  | Coverage area | | | | | | | | |
|  | Area (km^2^) | Percent | | Area (km^2^) | Percent | | Area (km^2^) | Percent | |
| *Min-0.1 | 35.7 | 8 | | 2.6 | < 1 | | - | - | |
| 0.1-0.2 | 113.1 | 22 | | 59.1 | 12 | | 3.6 | < 1 | |
| 0.2-0.5 | **189.3** | **37** | | **174.2** | **34** | | 102 | 20 | |
| 0.5-1 | 101.2 | 20 | | 138.4 | 27 | | **145** | **29** | |
| 1-2 | 46.6 | 9 | | 86.1 | 17 | | 134 | 26 | |
| 2-5 | 20.3 | 4 | | 39.3 | 8 | | 92.5 | 18 | |
| 5-10 | - | - | | 6.5 | 1 | | 24 | 5 | |
| 10-*Max | - | - | | - | - | | 4.6 | < 1 | |
| Weighted mean | 0.59 | | | 0.99 | | | 1.81 | | |
| **PM_10_** (µg/m^3^) | 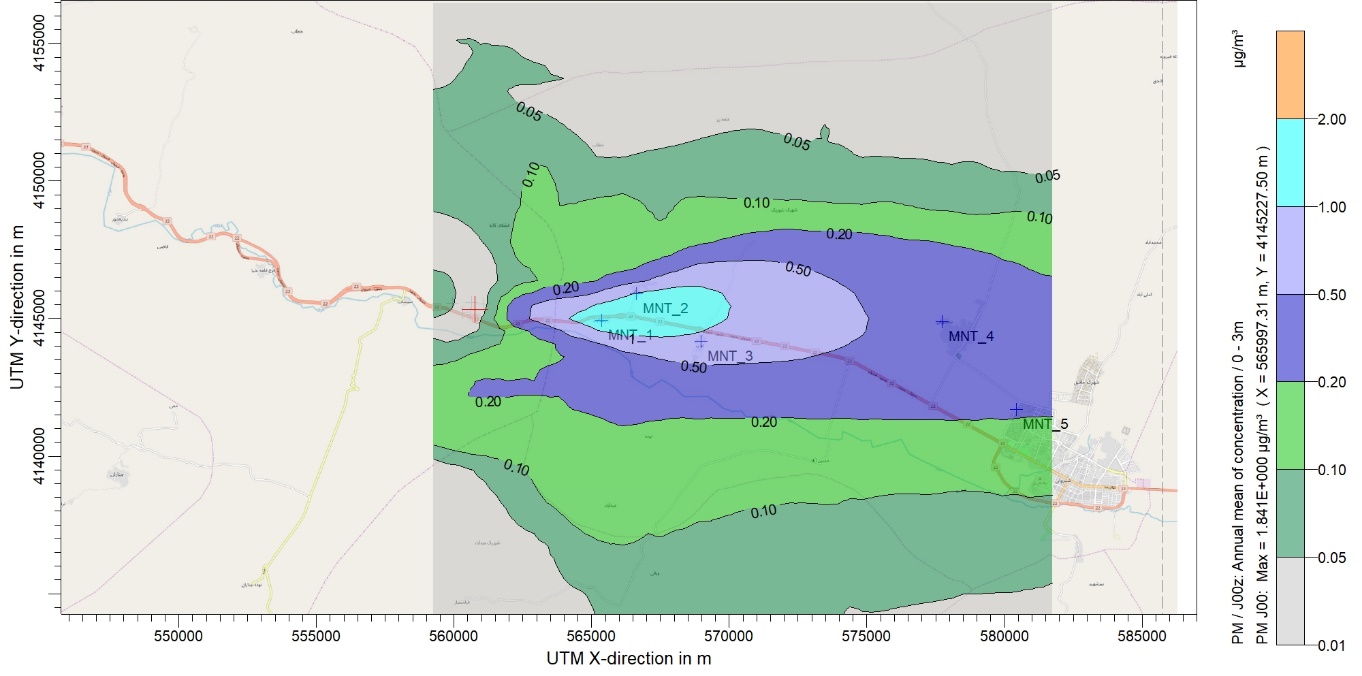 | | | 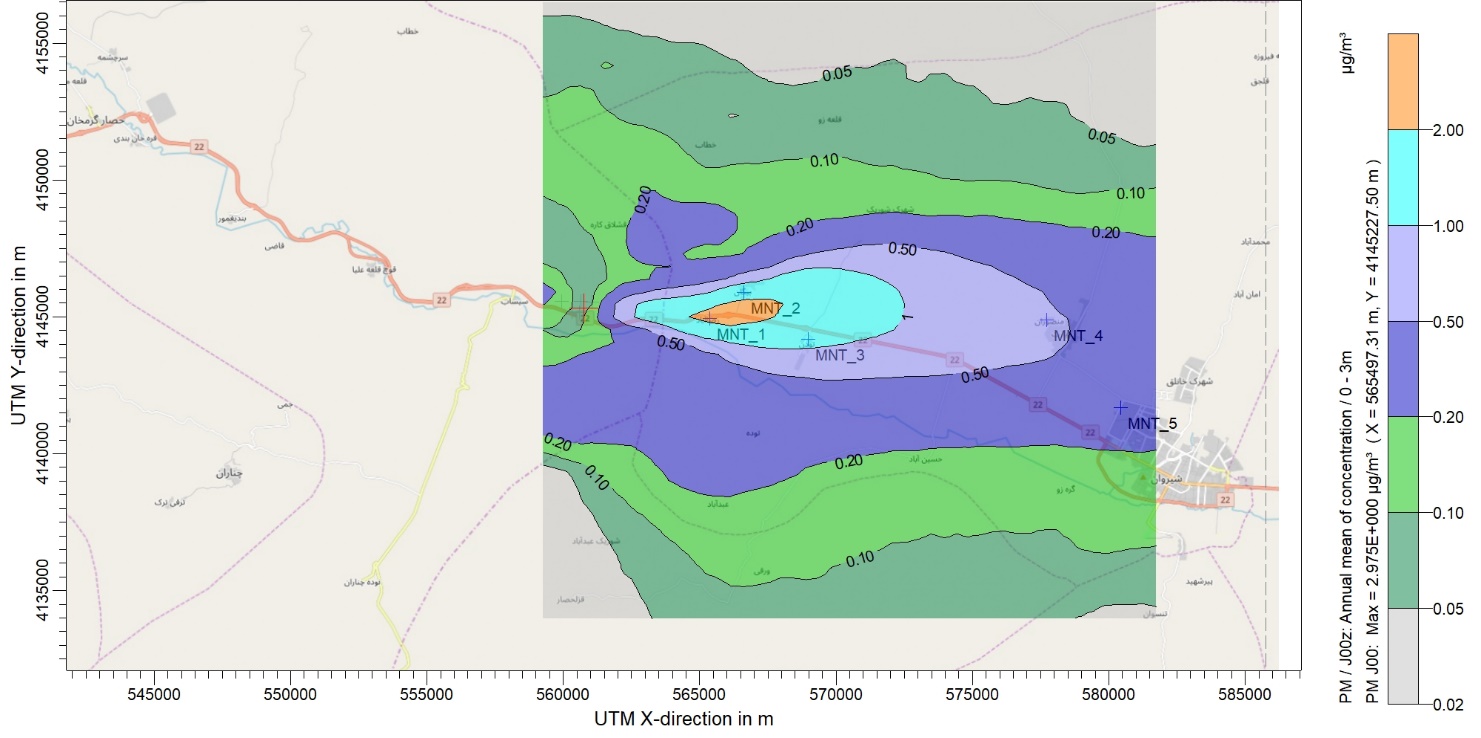 | | | 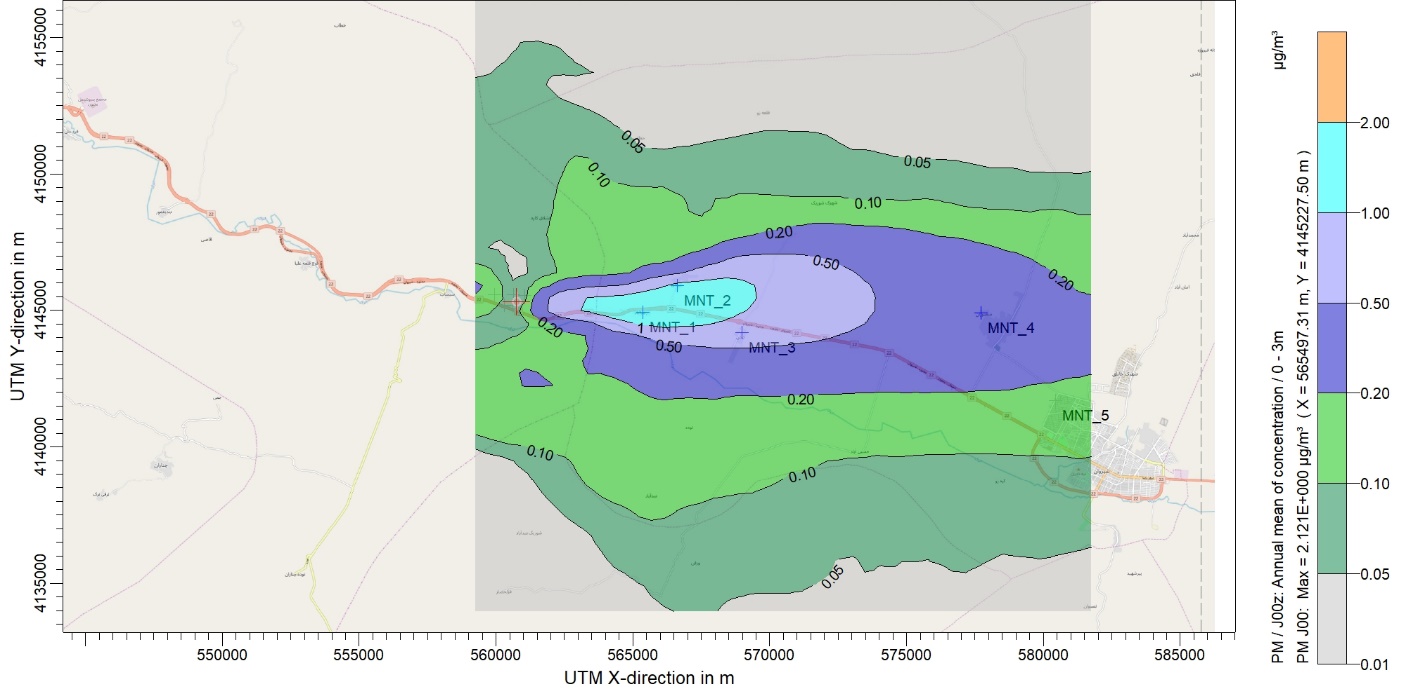 | | |
|  | Min= 0.01 , Max= 1.84 | | | Min= 0.02 , Max= 2.97 | | | Min= 0.01 , Max= 2.2 | | |
|  | Coverage area | | | | | | | | |
|  | Area (km^2^) | | Percent | Area (km^2^) | | Percent | Area (km^2^) | | Percent |
| Min-0.05 | **142.4** | | **28** | 73.7 | | 13 | **156.8** | | **31** |
| 0.05-0.1 | 135 | | 27 | **126.8** | | **25** | 135 | | 27 |
| 0.1-0.2 | 113.3 | | 22 | 124.1 | | 25 | 113.2 | | 22 |
| 0.2-0.5 | 82.7 | | 16 | 124.3 | | 25 | 72.5 | | 14 |
| 0.5-1 | 25.1 | | 5 | 37.4 | | 7 | 21 | | 4 |
| 1-2 | 7.5 | | 1 | 17.8 | | 4 | 7.7 | | 1 |
| 2-Max | - | | - | 2.1 | | < 1 | < 0.1 | | < 1 |
| Weighted mean | 0.17 | | | 0.28 | | | 0.17 | | |
| **SO_2_** (µg/m^3^) | 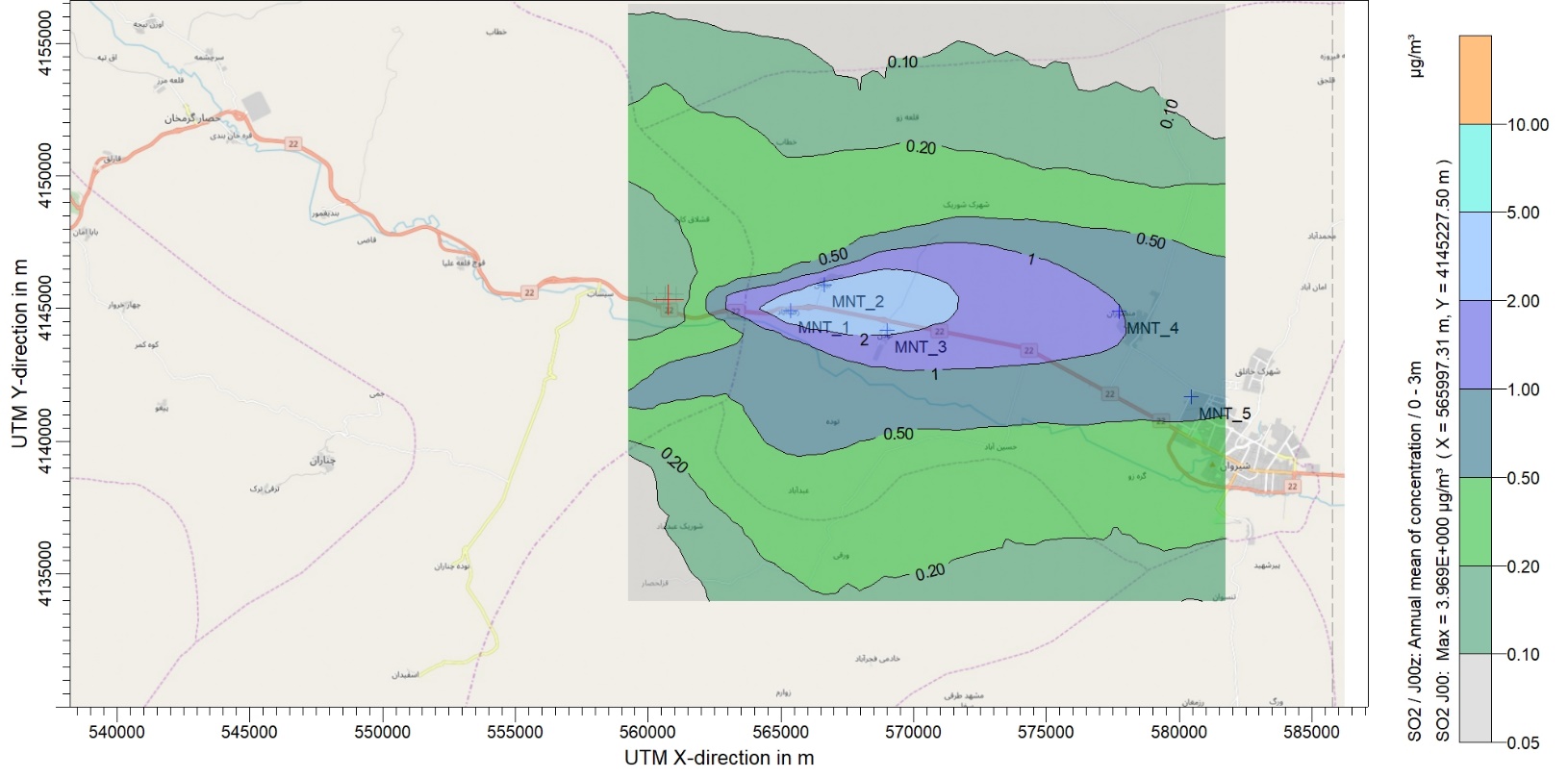 | | | 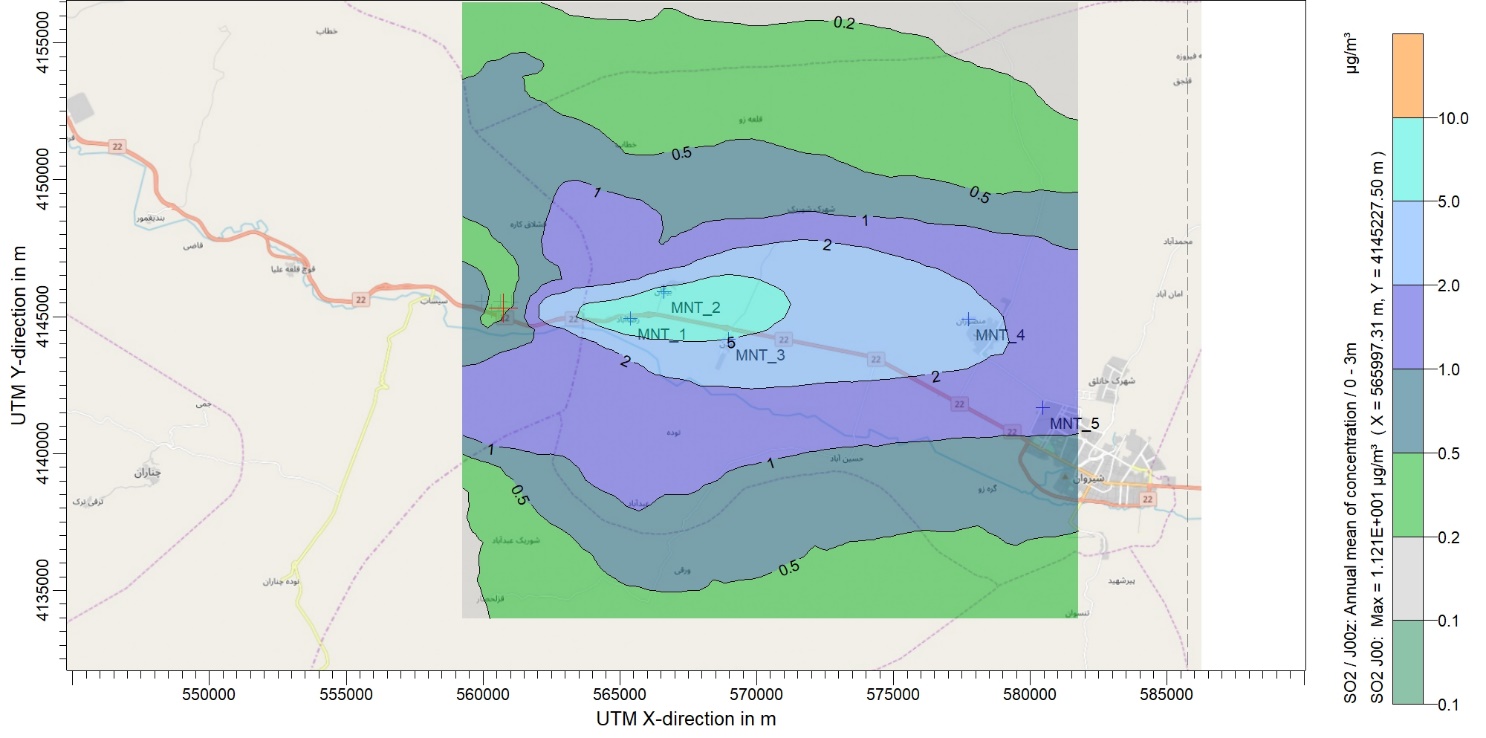 | | | 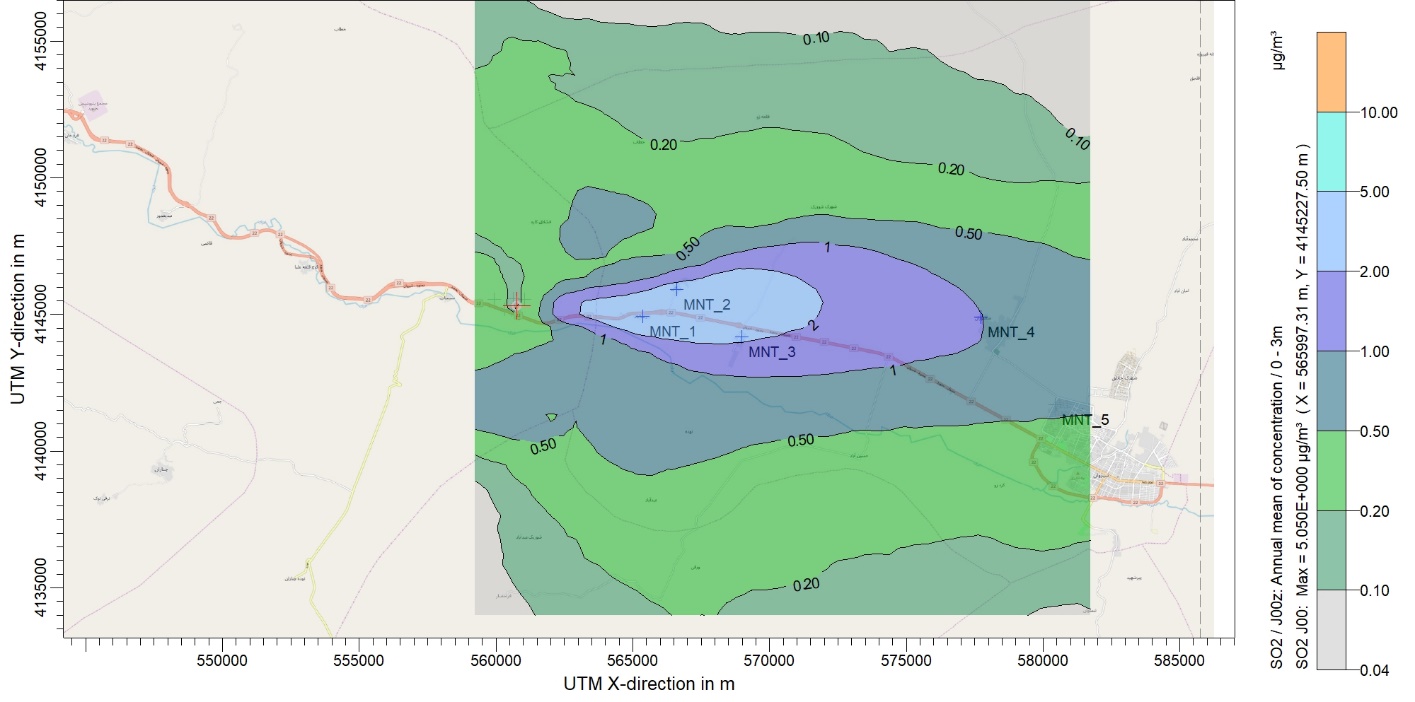 | | |
|  | Min= 0.04, Max= 3.96 | | | Min= 0.1, Max= 11.21 | | | Min= 0.04, Max= 5 | | |
|  | Coverage area | | | | | | | | |
|  | Area (km^2^) | Percent | | Area (km^2^) | Percent | | Area (km^2^) | Percent | |
| Min- 0.1 | 57.6 | 11 | | - | - | | 41.6 | 8 | |
| 0.1-0.2 | 127.6 | 25 | | 27.1 | 5 | | 119 | 24 | |
| 0.2-0.5 | **180.1** | **36** | | **158** | **31** | | **185.9** | **37** | |
| 0.5-1 | 89.8 | 18 | | 137.8 | 27 | | 105.6 | 21 | |
| 1-2 | 37.9 | 7 | | 118.1 | 23 | | 37.3 | 7 | |
| 2-5 | 13.1 | 3 | | 52 | 10 | | 16.6 | 3 | |
| 5-10 | - | - | | 13 | 3 | | - | - | |
| 10-Max | - | - | | < 0.1 | < 1 | | - | - | |
| Weighted mean | 0.51 | | | 1.34 | | | 0.53 | | |
| **CO** (g/m^3^) | 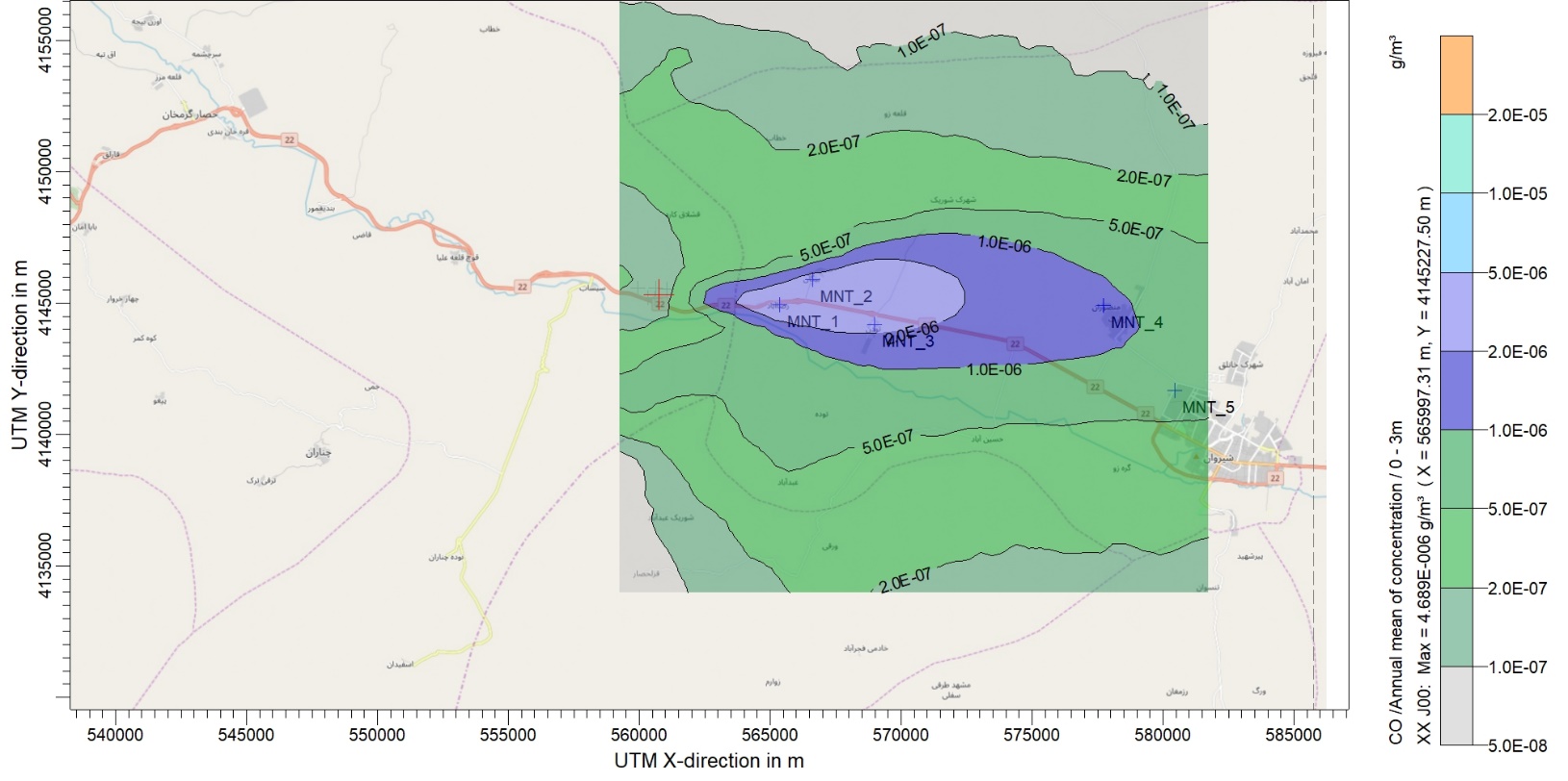 | | | 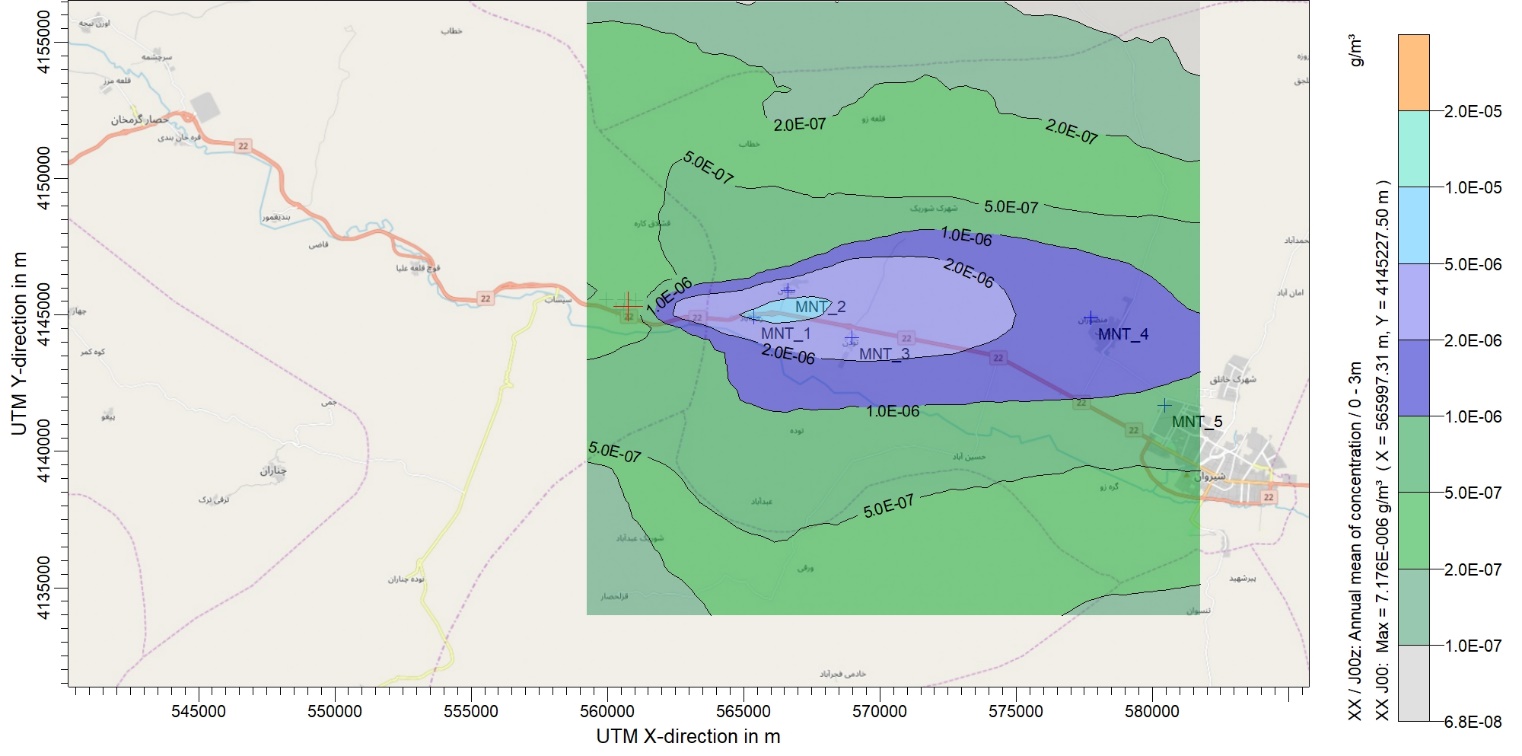 | | | 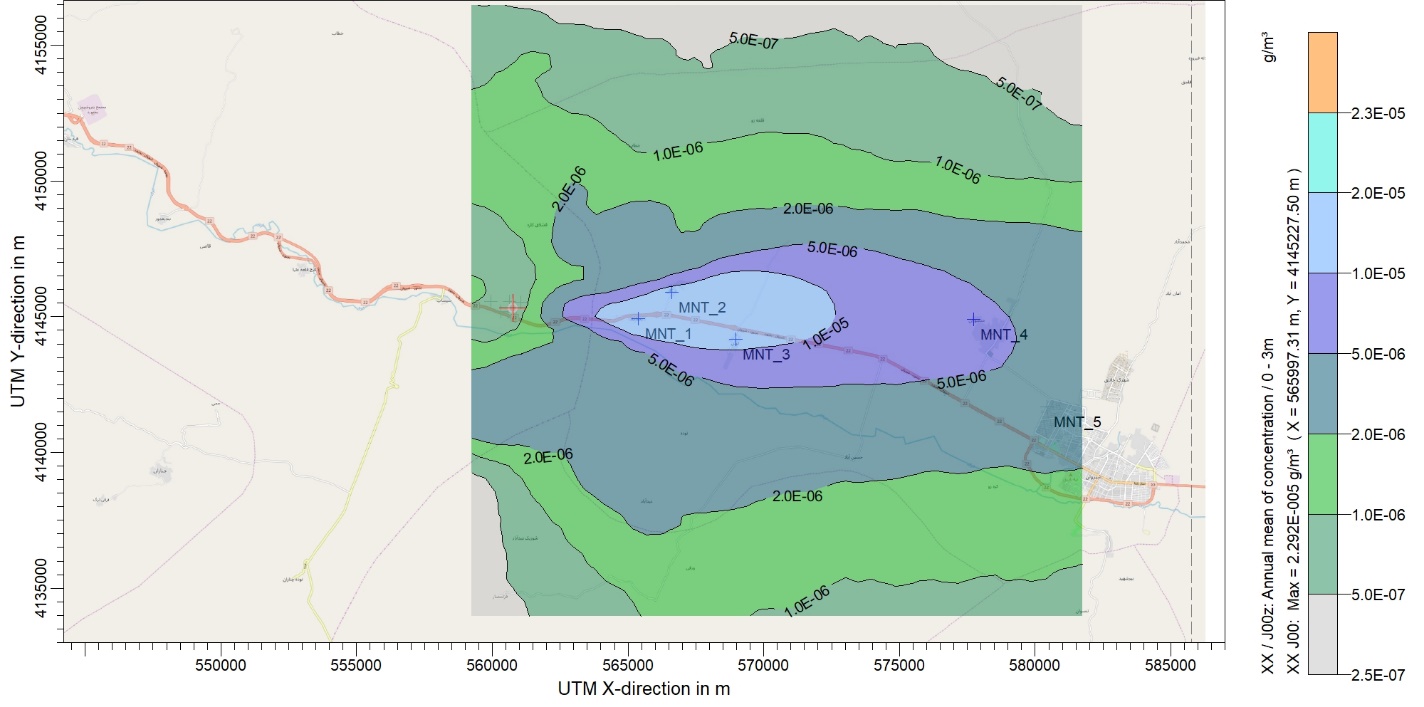 | | |
|  | Min= 0.05, Max= 4.68 | | | Min= 0.06 , Max= 7.17 | | | Min= 0.25, Max= 22.92 | | |
|  | Coverage area | | | | | | | | |
|  | Area (km^2^) | Percent | | Area (km^2^) | Percent | | Area (km^2^) | Percent | |
| Min-0.1 | 46.6 | 9 | | 7.9 | 2 | | - | - | |
| 0.1-0.2 | 119.8 | 24 | | 84.4 | 17 | | - | - | |
| 0.2-0.5 | **185.4** | **37** | | **187.6** | **37** | | 40.9 | 8 | |
| 0.5-1 | 94.5 | 19 | | 128.1 | 25 | | 114.1 | 23 | |
| 1-2 | 42.4 | 8 | | 64.7 | 13 | | 142.5 | 28 | |
| 2-5 | 17.3 | 3 | | 31.2 | 6 | | **145.7** | **29** | |
| 5-10 | - | - | | 2.13 | < 1 | | 44.6 | 9 | |
| 10-20 | - | - | | - | - | | 18.1 | 3 | |
| 20-Max | - | - | | - | - | | <0.1 | <1 | |
| Weighted mean | 0.53 | | | 0.82 | | | 2.77 | | |

Fig. 1s. Dispersion patterns of pollutants along the development periods

|  | **MNT 1^*^** | **MNT 2^*^** | **MNT 3^*^** | **MNT 4^*^** | **MNT 5^**^** |
| --- | --- | --- | --- | --- | --- |
| **Period 1** | 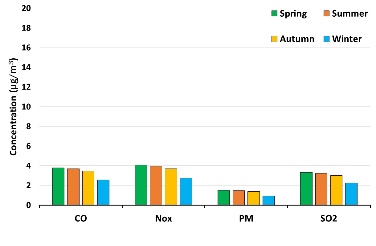 | 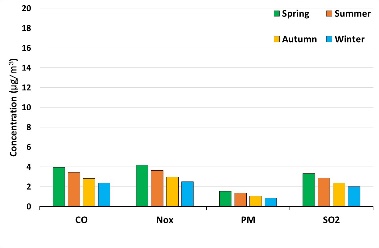 | 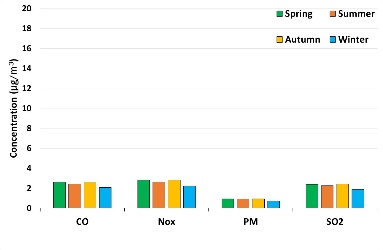 | 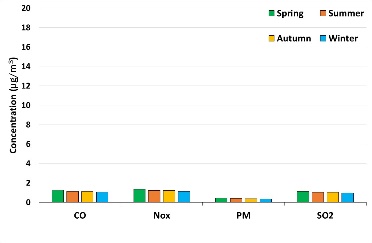 | 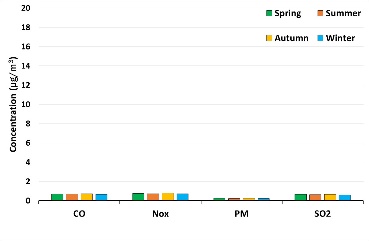 |
| **Period 2** | 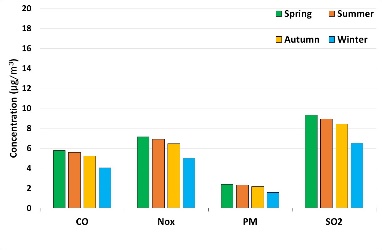 | 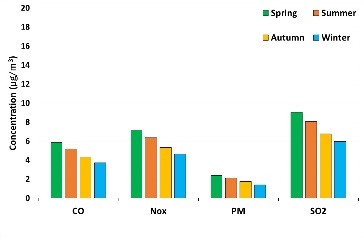 | 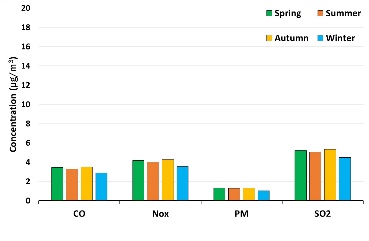 | 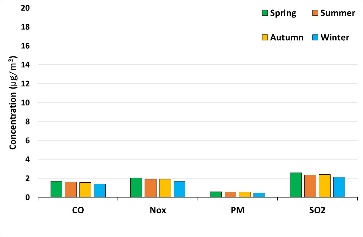 | 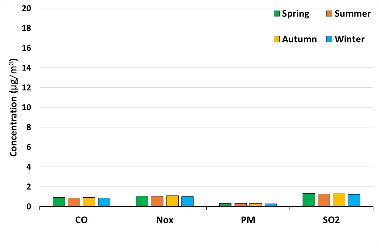 |
| **Period 3** | 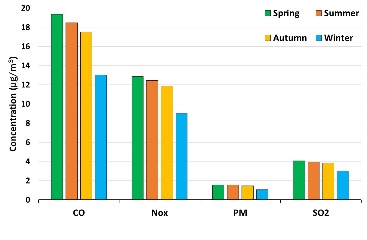 | 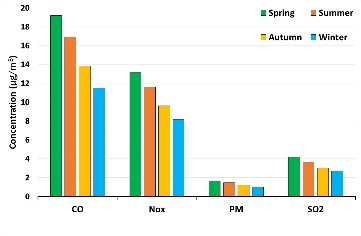 | 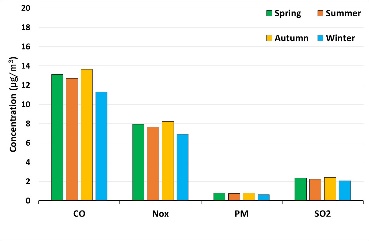 | 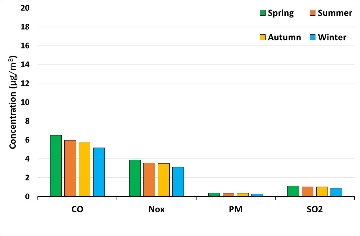 | 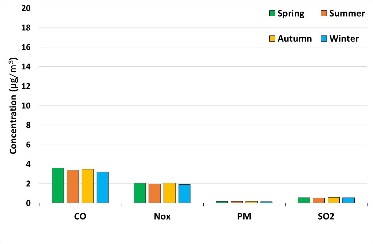 |

**^*^** Rural area

**^**^** Urban area

Fig. 2s. Seasonal prediction of pollutants concentration at monitoring points

| MNT-6 | CO | R^2^= -0.865 | 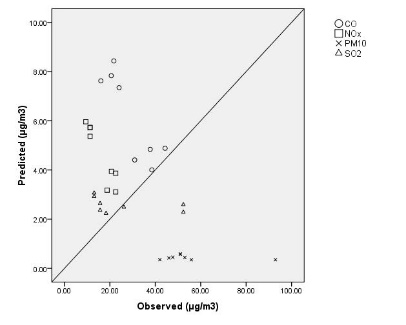 |
| --- | --- | --- | --- |
|  |  | P= 0.006 |  |
|  | NO_x_ | R^2^= -0.935 |  |
|  |  | P= 0.001 |  |
|  | PM_10_ | **R^2^= -0.320** |  |
|  |  | **P= 0.440** |  |
|  | SO_2_ | **R^2^= -0.399** |  |
|  |  | **P= 0.328** |  |
| MNT-7 | CO | **R^2^= 0.155** | 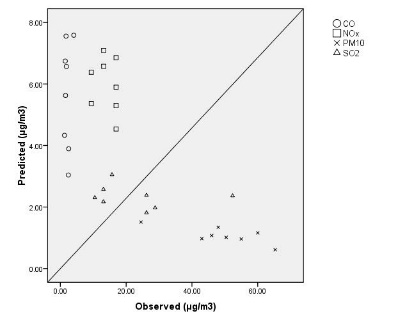 |
|  |  | **P= 0.714** |  |
|  | NO_x_ | **R^2^= -0.212** |  |
|  |  | **P= 0.614** |  |
|  | PM_10_ | R^2^= -0.759 |  |
|  |  | P= 0.029 |  |
|  | SO_2_ | **R^2^= -0.230** |  |
|  |  | **P= 0.583** |  |
| MNT-8 | CO | R^2^= 0.708 | 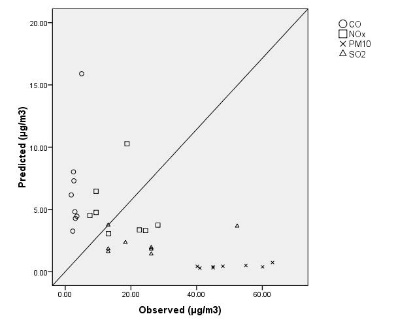 |
|  |  | P= 0.049 |  |
|  | NO_x_ | **R^2^= -0.177** |  |
|  |  | **P= 0.676** |  |
|  | PM_10_ | **R^2^= 0.706** |  |
|  |  | **P= 0.050** |  |
|  | SO_2_ | **R^2^= 0.377** |  |
|  |  | **P= 0.358** |  |
| MNT-9 | CO | **R^2^= 0.288** | 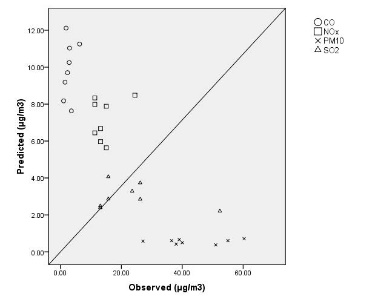 |
|  |  | **P= 0.489** |  |
|  | NO_x_ | **R^2^= 0.316** |  |
|  |  | **P= 0.446** |  |
|  | PM_10_ | **R^2^= 0.193** |  |
|  |  | **P= 0.647** |  |
|  | SO_2_ | **R^2^= -0.279** |  |
|  |  | **P= 0.503** |  |
| MNT-10 | CO | R^2^= -0.710 | 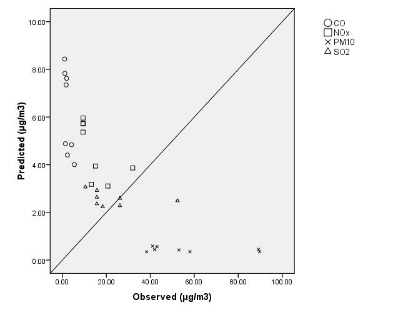 |
|  |  | P= 0.049 |  |
|  | NO_x_ | **R^2^= -0.633** |  |
|  |  | **P= 0.092** |  |
|  | PM_10_ | **R^2^= -0.366** |  |
|  |  | **P= 0.373** |  |
|  | SO_2_ | **R^2^= -0.344** |  |
|  |  | **P= 0.404** |  |

Fig. 3s. Q-Q plot of the observed and predicted pollutants concentration at specified monitoring points
